# Photon-counting detector computed tomography in cardiac imaging

**DOI:** 10.1007/s12471-024-01904-5

**Published:** 2024-10-02

**Authors:** Simran P. Sharma, Marie-Julie D. K. Lemmens, Martijn W. Smulders, Ricardo P. J. Budde, Alexander Hirsch, Casper Mihl

**Affiliations:** 1 Department of Radiology and Nuclear Medicine, Erasmus Medical Centre, University Medical Centre, Rotterdam, The Netherlands; 2 Department of Cardiology, Cardiovascular Institute, Erasmus Medical Centre, University Medical Centre, Rotterdam, The Netherlands; 3 Cardiovascular Research Institute Maastricht (CARIM), Maastricht University, Maastricht, The Netherlands; 4 Department of Cardiology, Maastricht University Medical Centre, Maastricht, The Netherlands; 5 Department of Radiology and Nuclear Medicine, Maastricht University Medical Centre, Maastricht, The Netherlands

**Keywords:** Coronary artery disease, Cardiac imaging techniques, Tomography, X‑ray computed, Angiography

## Abstract

Photon-counting detector computed tomography (PCD-CT) has emerged as a revolutionary technology in CT imaging. PCD-CT offers significant advancements over conventional energy-integrating detector CT, including increased spatial resolution, artefact reduction and inherent spectral imaging capabilities. In cardiac imaging, PCD-CT can offer a more accurate assessment of coronary artery disease, plaque characterisation and the in-stent lumen. Additionally, it might improve the visualisation of myocardial fibrosis through qualitative late enhancement imaging and quantitative extracellular volume measurements. The use of PCD-CT in cardiac imaging holds significant potential, positioning itself as a valuable modality that could serve as a one-stop-shop by integrating both angiography and tissue characterisation into a single examination. Despite its potential, large-scale clinical trials, standardisation of protocols and cost-effectiveness considerations are required for its broader integration into clinical practice. This narrative review provides an overview of the current literature on PCD-CT regarding the possibilities and limitations of cardiac imaging.

## Introduction

**Fig. 1:**
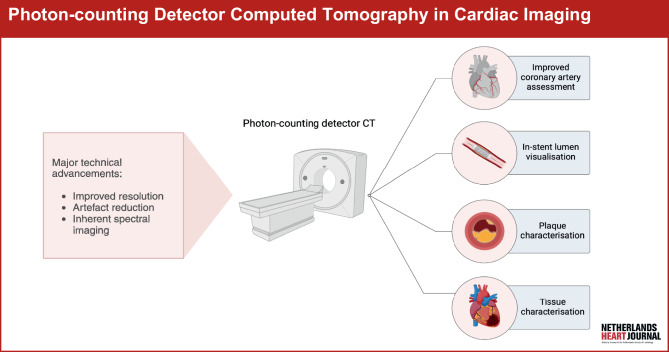
Infographic: Photon-counting detector computed tomography in cardiac imaging

**Fig. 2:**
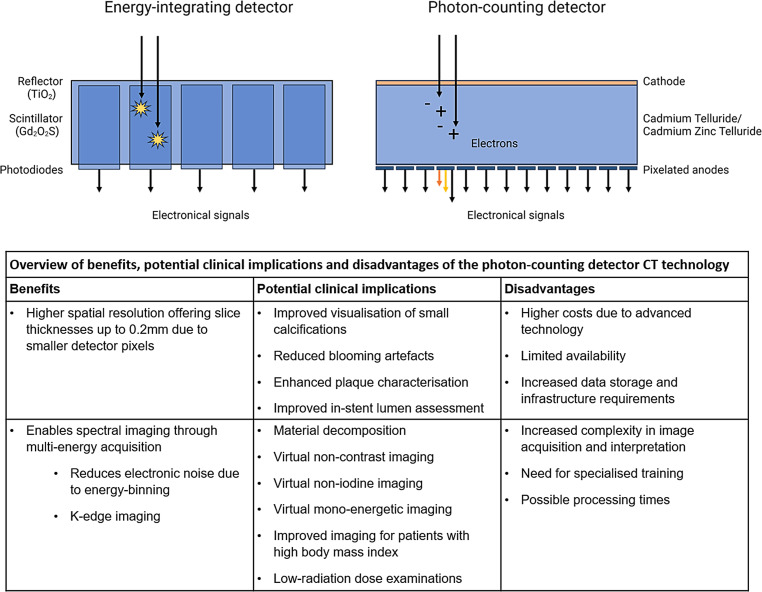
Overview of energy-integrating versus photon-counting detector. The energy-integrating detector uses a scintillator (e.g. gadolinium oxysulfide) to absorb X‑rays and convert them into visible light, which is then detected and converted into electrical signals by photodiodes. The photon-counting detector (PCD) uses a Cadmium Telluride/Cadmium Zinc Telluride or Silicon detector to directly convert incoming X‑rays into a charge cloud of electrons, which are collected by pixelated anodes. The electronic signals’ varying colours indicate the different energy levels of the photons, highlighting PCD’s energy-resolving capability

Photon-counting detector computed tomography (PCD-CT) is a new CT imaging technique that will likely reshape the cardiac imaging landscape (Fig. 1; [1]). The primary innovation of PCD-CT lies in its detector technology, which significantly differs from that used in conventional energy-integrating detectors (EID-CT) [1–3]. Figure 2 provides a schematic overview of EID and PCD technology and an overview of the benefits, alongside their potential clinical implications, and the disadvantages of PCD-CT.

A key feature of PCD-CT technology is the use of smaller detector elements which enhances spatial resolution. Consequently, it facilitates high-resolution imaging with slice thicknesses down to 0.2 mm, while EID-CT systems typically offer slice thicknesses of 0.6 mm [4]. Secondly, PCD-CT enhances spectral imaging beyond what is achievable with conventional CT systems [5]. By counting individual incoming photons and measuring their energy, the detector can categorise these photons into various energy bins. This process allows better X‑ray photon energy separation compared with conventional EID-based dual-energy CT, improving differentiation of tissue types and materials. It also facilitates advanced image reconstructions including virtual non-iodine (VNI) images and virtual non-calcium images derived from contrast-enhanced scans, as well as virtual mono-energetic images (VMIs) [5]. For a detailed description of the principles of spectral imaging, we refer to a comprehensive review by Danad et al. [6].

All major vendors are currently developing PCD-CT systems and have reported on applications in humans [7–9]. However, only one system (NAEOTOM Alpha, Siemens Healthineers) is currently commercially available [3].

In line with these advancements, the adoption of PCD-CT is becoming increasingly prominent in cardiac imaging. In the Netherlands, this development is particularly evident now the commercially available PCD-CT systems (NAEOTOM Alpha, Siemens Healthineers) are installed or being installed, along with the upcoming introduction of another prototype scanner from Canon Inc. Reflecting the growing interest in PCD technology within the Dutch radiology and cardiology community, this review elaborates on the specific applications, potential benefits and limitations of PCD-CT in the context of cardiac diseases.

## Coronary artery disease

### Coronary calcium scoring

Coronary artery calcium (CAC) scoring, typically quantified using the Agatston method on non-enhanced CT scans (with a slice thickness of 2.5–3 mm), serves as an indicator of coronary atherosclerosis and predicts cardiovascular events [10, 11]. A phantom study by Van der Werf et al. demonstrated that CAC scores obtained through routine clinical scan protocols are similar between conventional CT and PCD-CT [12]. Notably, the increased spatial resolution of PCD-CT facilitates improved detectability and more accurate estimation of CAC volumes, especially at reduced slice thicknesses (< 1 mm) [12]. This enhanced resolution may allow the detection of even minute calcifications. However, with the ongoing advancements in CT technology, an important consideration that arises is the clinical relevance of identifying very small calcifications and how this affects patient management and outcomes.

In addition to enhanced detectability, PCD-CT technology could potentially obviate the need for non-contrast scans to determine CAC scores. The ability to perform CAC scoring based on contrast-enhanced coronary CT angiography (CCTA) offers benefits such as reduced scan time and an expected decrease in radiation dose, especially since CAC scoring usually requires a higher kV setting. With the introduction of spectral imaging techniques, various studies have explored algorithms for estimating CAC scores from virtual non-contrast (VNC) images derived from contrast-enhanced images, which are generated from two-material decomposition into water and iodine [13–15]. Nonetheless, this approach has been consistently found to underestimate CAC scores and even cause false negatives, raising concerns about its clinical utility [13, 14].

To address this limitation, a novel algorithm was introduced that removes iodine from the contrast-enhanced scans, leaving only calcium in the resulting VNI images [16]. A patient study conducted by Emrich et al. revealed that VNI images outperform VNC images in the accuracy of CAC quantification; however, a significant underestimation of CAC scores persisted in VNI reconstructions [16]. Similarly, Sharma et al. reported a superior performance of VNI reconstructions compared with VNC [17]. Conversely, in their study, VNI reconstructions overestimated the CAC scores. Discrepancies in results may stem from variances in software versions and reconstruction methods for VNC and VNI images.

**Fig. 3:**
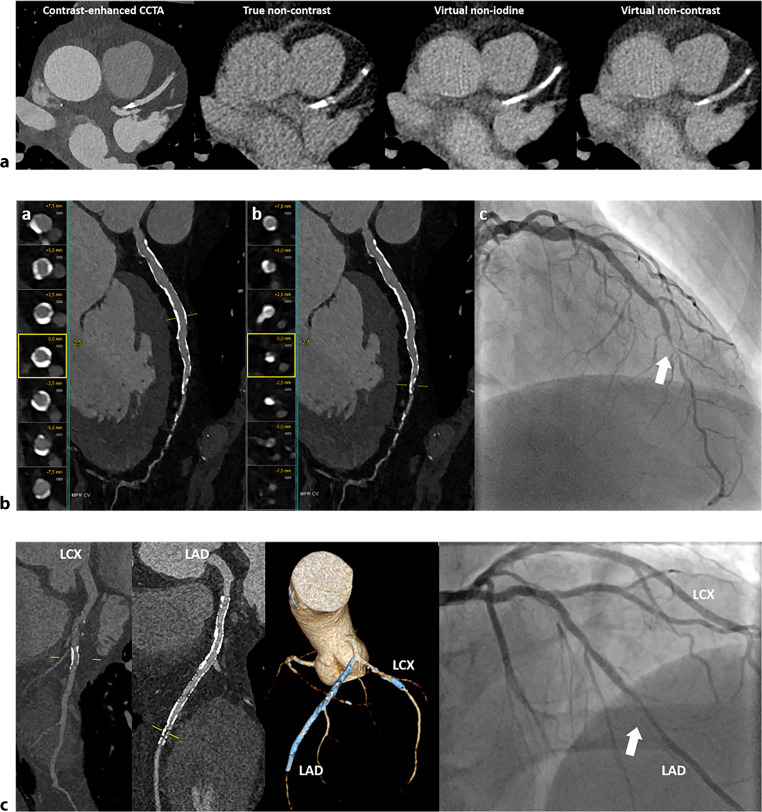
Advanced coronary artery disease assessment using photon-counting detector CT (PCD-CT). **a** Calcium artery calcium scoring using PCD-CT on contrast-enhanced, true non-contrast, virtual non-iodine and virtual non-contrast images. The calcified plaque in the left anterior descending (LAD) artery appears more pronounced on the virtual non-iodine image and less pronounced on the virtual non-contrast image, relative to the true non-contrast image. **b** Ultra-high resolution imaging of coronary arteries in a 66-year-old man with stable angina and multiple risk factors. (a) Multiplanar reformatted (MPR) images of the LAD, depicting extensive calcification of the vessel wall. MPR and cross-sectional views reveal no significant stenosis in the proximal and mid-LAD. (b) MPR images of the LAD with cross-sectional views illustrate significant lumen tapering in the transition from the mid to distal LAD. (c) Invasive coronary angiography confirms the presence of significant stenosis in the mid/distal LAD. **c** Stent-imaging using ultra-high resolution PCD-CT. Coronary CT angiography of a 67-year-old male patient with typical chest pain was performed using PCD-CT in ultra-high resolution mode. The patient has a history of stent placement in the LAD and left circumflex (LCX) artery. Multiplanar reconstruction images display a patent stent in the LCX. However, in the LAD, there is evidence of in-stent restenosis located distally within the stent. The 3D volume rendering clearly depicts the stents in both the LAD and LCX. Corresponding invasive coronary angiography confirms the presence of in-stent restenosis in the LAD and the patent stent in the LCX

Currently, neither VNC nor VNI reconstructions are reliable enough to completely replace traditional non-contrast acquisition for CAC scoring. While VNI reconstructions might eventually eliminate the need for non-contrast enhanced calcium scoring, further optimisation is necessary for the clinical implementation of these algorithms. Figure 3a illustrates CAC scoring using PCD-CT.

### Coronary artery assessment

CCTA has emerged as a pivotal first-line diagnostic test for patients suspected of coronary artery disease [11]. However, the accurate quantification of coronary stenosis remains a challenge, particularly in patients with extensive or dense coronary artery calcifications. These calcifications can lead to blooming artefacts, often resulting in overestimated stenosis severity and false-positive diagnoses [18]. A previous meta-analysis demonstrated that CCTA, on a per-patient level, achieved a pooled sensitivity of 99%, specificity of 89%, median positive predictive value of 93% (range 64–100%), and negative predictive value of 100% (range 86–100%) for detecting significant stenosis as defined by invasive coronary angiography (ICA) [19].

The increased spatial resolution, along with the maintained high temporal resolution provided by dual-source PCDs, plays a crucial role in reducing blooming artefacts. This high-resolution capability, even allowing an ultra-high resolution (UHR) mode, is a defining feature of PCD-CT technology, enabling more detailed and precise imaging than is possible with conventional CT systems. Although slice thickness varies between systems and resolution modes, it is thinner (down to 0.2 mm) than that achieved with conventional EID-CT [1, 3, 4, 20]. In the UHR mode, it is recommended to employ dedicated sharp kernels for optimal visualisation of coronary plaques and the vessel lumen [21]. Figure 3b presents an example of UHR imaging.

In an initial study involving 14 patients, the UHR mode of PCD-CT enabled improved image quality and diagnostic confidence for CCTA examinations at a comparable dose, in comparison with conventional EID-CT [7]. Furthermore, a phantom study indicated that UHR CCTA significantly reduces blooming artefacts and improves stenosis quantification compared with standard resolution CCTA, indicating enhanced accuracy [22]. A head-to-head comparison between EID-CT and UHR PCD-CT in patients with dense coronary calcifications was carried out in the study by Koons et al. [23]. This research demonstrated a decrease in percent diameter stenosis by an average of 11% due to the reduction of blooming artefacts with the use of UHR PCD-CT compared with EID-CT. As a result, 13 out of 34 stenoses were downgraded in the stenosis severity category based on the Coronary Artery Disease Reporting and Data System. Specifically, nine were downgraded from mild to minimal, three from moderate to minimal, and one from moderate to mild. In addition, two were upgraded from minimal to mild. It is important to note that this comparison did not include a reference standard such as ICA.

While the UHR mode in PCD-CT enhances spatial resolution, an equally important feature of PCD-CT technology is its inherent spectral imaging capabilities [1]. The study by McCollough et al. compared standard resolution PCD-CT, utilising its spectral capabilities, with EID-CT. Their results showed that PCD-CT at 100 keV effectively reduced calcium blooming compared with EID-CT, resulting in a decrease in the visual estimates of percent diameter luminal stenosis [24]. The spectral capabilities of PCD-CT also offer a calcium-removal reconstruction algorithm, which enables lumen evaluation without the influence of blooming artefacts. The study by Nishihara et al. demonstrated that the calcium-removal image algorithm using PCD-CT improved diagnostic accuracy over conventional images and enhanced image interpretability of severely calcified coronary lesions [25].

A recent study by Hagar et al. focused on evaluating the diagnostic accuracy of UHR PCD-CT in detecting coronary artery disease in patients with severe aortic valve stenosis referred for transcatheter aortic valve replacement (TAVR) [26]. Using ICA as the reference standard, this prospective study involved 68 participants with a median CAC score of 414 (25th–75th percentile: 125–1246). The results demonstrated a sensitivity of 96%, specificity of 84%, positive predictive value of 77%, negative predictive value of 97%, and overall accuracy of 88% in the detection of stenosis of ≥ 50%.

Given the current evidence indicating that PCD-CT can effectively decrease blooming artefacts and enhance stenosis assessment accuracy, this technology has the potential to reduce false-positives in coronary artery disease diagnosis. Building on the already high negative predictive value of CCTA, PCD-CT might also improve specificity, even in patients with severe calcifications. This improvement could lead to a decrease in both unnecessary ICAs and repeated scans, providing a more patient-friendly approach and ultimately offering potential cost benefits while reducing radiation exposure [27].

### Functional assessment of coronary stenosis

Functional assessment of coronary stenosis through CT-derived fractional flow reserve (FFR_CT_) offers another promising approach for reducing unnecessary ICAs. The enhanced visualisation of coronary stenosis and reduction in blooming artefacts observed with PCD-CT may directly impact FFR_CT_ computation. Zsarnoczay et al. demonstrated the feasibility of FFR_CT_ with PCD-CT, reporting excellent correlation and strong agreement with conventional EID-CT [28]. Furthermore, in a study involving TAVR patients, Brendel et al. found that the integration of PCD-CT, combined with artificial intelligence-derived CCTA stenosis quantification and FFR_CT_, could potentially obviate the need for ICA in 121 out of 260 patients (46.5%) [27]. These findings underscore the potential of FFR_CT_ in combination with PCD-CT to optimise patient management strategies by reducing unnecessary invasive procedures. This is especially relevant in frail patients such as the TAVR population.

### Stent imaging

The assessment of in-stent restenosis remains challenging in conventional CT due to blooming and beam-hardening artefacts. However, PCD-CT shows promise in enhancing the sensitivity and specificity of CT for patients with prior stent placement [29, 30]. Key to this improvement is the selection of appropriate acquisition and reconstruction parameters tailored for stent imaging. UHR imaging, particularly when combined with sharp stent-specific kernels at a tube voltage of either 120 or 140 kV, appears to be the most effective approach [31]. At present, the high resolution provides the greatest added value for evaluating the stent lumen. Nevertheless, the integration of spectral imaging with the UHR mode promises to further enhance diagnostic capabilities for stent evaluation.

In a study by Hagar et al., UHR PCD-CT was used to evaluate coronary stent patency in 18 participants with 44 stents, demonstrating high diagnostic accuracy (89%) compared with ICA, which relied on visual assessment [32]. The study reported excellent sensitivity (100%) and specificity (87%), high negative predictive (100%) value and substantial inter-reader agreement, indicating the potential of UHR PCD-CT in stent assessment. While these findings are promising, more evidence is needed before PCD-CT can be considered a definitive alternative to ICA for ruling out in-stent stenosis. Figure 3c displays an example of stent imaging with PCD-CT.

## Myocardial fibrosis

### Assessment of myocardial fibrosis in current practice

To date, late enhancement (LE) with gadolinium in cardiovascular magnetic resonance (CMR) imaging is considered the reference standard for the non-invasive assessment of myocardial infarction, scar and viability [33]. The application of LE imaging in CMR (LE_CMR_) is not only confined to the evaluation of infarction but is also useful in any type of pathology causing focal myocardial fibrosis [34]. The principle of LE imaging is based on the accumulation and retainment of contrast agents in an enlarged extracellular space [35, 36]. In patients with acute or chronic myocardial injury, this extracellular volume (ECV) is enlarged, either by oedema and cardiomyocyte membrane rupture, or by replacement fibrosis. Focal fibrosis is commonly assessed visually or semi-quantitively, while diffuse fibrosis is measured quantitatively using native T1 and ECV mapping [37]. LE_CMR_ and T1/ECV mapping enable differentiation between ischaemic and non-ischaemic diseases such as sarcoidosis, amyloidosis, dilated cardiomyopathy and myocarditis by visualising typical LE_CMR_ patterns and native T1 and ECV maps [36, 38]. Furthermore, the presence and extent of fibrosis on LE_CMR_ may change therapy and is highly correlated to prognosis [38].

### Late enhancement imaging with CT

Technological advances have paved the way for visualising fibrosis with CT in addition to CMR. A study by Andreini et al. revealed a high diagnostic concordance of 94.7% between LE_CMR_ and LE with CT (LE_CT_) patterns [39].

**Fig. 4:**
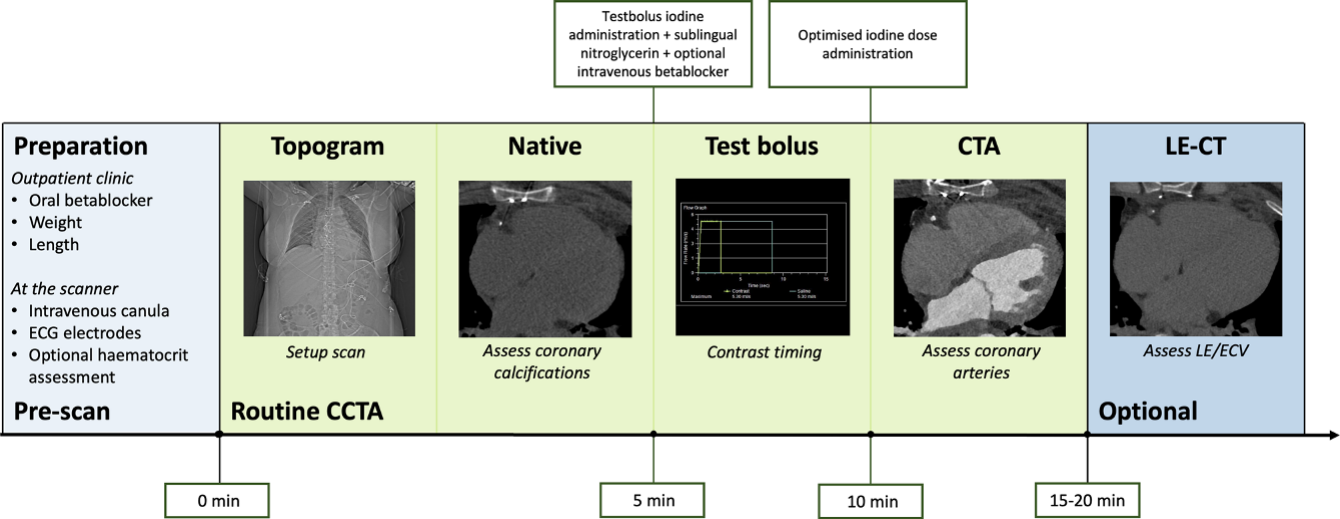
Photon-counting detector CT imaging protocol as a one-stop-shop for both coronary artery assessment and late enhancement imaging. The duration of a regular coronary CT angiography (CCTA) protocol is approximately 10 min, an additional late enhancement (LE) CT acquisition extends the total duration to approximately 15–20 min. Acquisition times may differ between centres. A haematocrit assessment is only necessary for the optional extracellular volume (ECV) assessment acquired 5–10 min after contrast administration. A native scan is optional as it can be reconstructed as a virtual non-iodine image

The introduction of LE_CT_ in clinical settings may be favourable as it encompasses several advantages. By combining CCTA and LE_CT_ in a single examination, CT could serve as a one-stop-shop assessing both coronary artery disease and myocardial enhancement patterns. This allows not only differentiation between ischaemic and non-ischaemic cardiomyopathy, but also the assessment of viability in the case of ischaemic cardiomyopathy using a single modality. In addition, important contraindications for CMR, such as claustrophobia, non-MRI-conditional cardiac devices or ferromagnetic implantations, do not apply to CT. In general, CT scanning is readily available with shorter scanning times, while CMR may suffer from longer waiting lists, longer acquisition times and higher costs. Nevertheless, incorporating LE_CT_ into its scan protocol does lengthen CT acquisition time as well, which is also accompanied with an increased radiation and at times a higher contrast dose. An example of a combined CCTA and LE_(PCD-)CT_ scan protocol is described in Fig. 4.

Multiple studies have proposed LE_CT_ imaging in 3rd generation dual-energy CT scanners as an adequate alternative, benefiting from higher spatial resolution when compared with CMR and surpassing the discriminatory capacities of single-energy CT scanners [40]. However, CMR remains the reference standard as the implementation of LE_CT_ is mostly constrained by a relatively low contrast-to-noise ratio [41]. PCD-CT might improve this issue, benefiting from a substantially higher spatial resolution and improved contrast-to-noise ratio [42]. An initial case study with LE_PCD-CT_ has shown hyper-enhancement patterns remarkably similar to the pattern visualised with LE_CMR_ [43].

Multi-energy acquisition could augment the versatility of PCD-CT to an even greater extent, as this approach facilitates the separation of multiple contrast agents when administered simultaneously [1, 44]. Symons et al. have demonstrated the feasibility of this concept in an occlusion-reperfusion canine model of myocardial infarction [45]. By combining iodine, gadolinium and soft tissue maps in PCD-CT, infarcted myocardium could be adequately distinguished from blood pool and normal myocardial tissue, in comparison with CMR and histopathology [45]. This could in theory facilitate the simultaneous assessment of coronary arteries (using iodine) and fibrosis (using gadolinium) within the same scan.

### Extracellular volume measurements with CT

Derived from CMR native and post-contrast T1 mapping, quantitative analysis with ECV_CMR_ calculations reflects the extent of the extracellular space [33]. This technique provides essential additional information, not only due to its ability to quantify the magnitude of fibrosis but also in the detection of diffuse fibrosis and early subtle fibrotic changes not readily visible with LE_CMR_ imaging [41].

ECV imaging with cardiac CT (ECV_CT_) has been compared with ECV_CMR_ in assessing diffuse myocardial fibrosis by several studies, hereby providing an appealing alternative to CMR [41]. A recent meta-analysis of 17 studies showed good agreement and excellent correlation of ECV_CT_ when compared with ECV_CMR_ [46]. ECV_CT_ can be calculated using the same principle as ECV_CMR_, but instead of differences in T1 signal, it relies on differences in attenuation, measured in Hounsfield units [41]. With dual-energy CT, the iodine-density derived method for ECV_CT_ calculation uses generated iodine-specific images and does not require non-contrast data [40].

ECV_CMR_ measurements previously demonstrated their clinical significance in terms of prognostic correlations [47]. Similar trends have been reported in ECV_CT_ measurements. Elevated ECV_CT_ has been associated with an increased risk of heart failure hospitalisation, major adverse cardiac events, and/or all-cause mortality in post-intervention cases and multiple cardiovascular diseases [48–50].

**Fig. 5:**
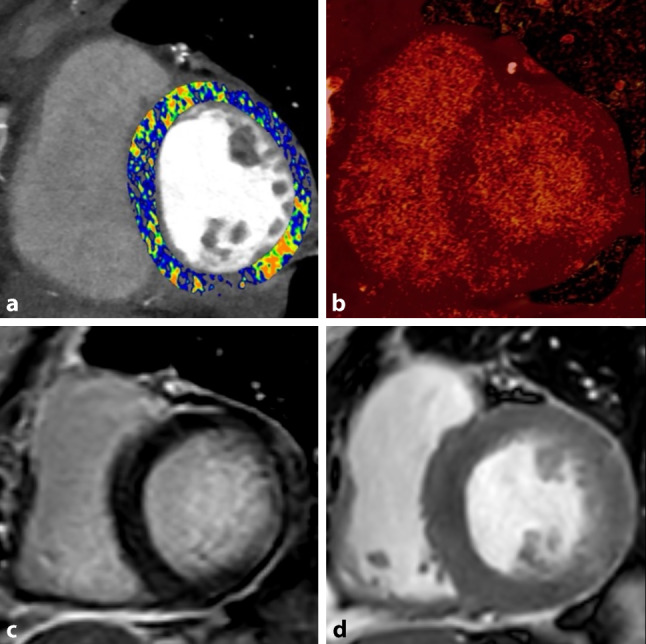
Short-axis photon-counting detector CT (PCD-CT, **a** and **b**) and cardiovascular magnetic resonance images (**c** and **d**) of a 59-year-old female recently diagnosed with myocardial infarction. *Image ***a** shows a PCD-CT extracellular volume (ECV) map of the left ventricle with focal increased ECV in the basal inferolateral segment. *Image ***b** shows a calculated iodine map with retainment of iodine in an ischaemic pattern, corresponding with an area of late gadolinium enhancement on cardiovascular magnetic resonance (*Image* **c**). *Image*** d** shows a short-axis cine image (after gadolinium administration) in which increased signal intensity and regional wall motion abnormalities were observed in the basal inferolateral segment, indicative of myocardial infarction

A recent study by Mergen et al. confirmed the feasibility of ECV measurements in PCD-CT (ECV_PCD-CT_) from the iodine ratio of LE scans in 30 patients with severe aortic stenosis undergoing PCD-CT for pre-procedural planning [51]. However, measurements were not compared with ECV_CMR_ measurements. In contrast, Aquino et al. did match PCD-CT findings with CMR in 29 patients undergoing CMR for several cardiovascular indications, showing strong correlations (*r* = 0.82–0.91, *p* < 0.001) and excellent reliability (intraclass correlation coefficient 0.81–0.90) between ECV_PCD-CT_ and ECV_CMR_ [52]. These findings suggest that ECV_PCD-CT_ could serve as an adequate alternative for ECV_CMR_ measurements in cardiovascular imaging. Figure 5 presents an example of LE_PCD-CT_ and ECV_PCD-CT_ in comparison with LE_CMR_ images.

## Future directions and challenges

PCD-CT has emerged as a novel imaging technique with promising results, especially in cardiac imaging. Alongside the previously stated applications, several other possibilities may be considered.

One of these possibilities is improved quantitative plaque characterisation with PCD-CT. The UHR mode of PCD-CT has demonstrated lower total plaque volumes and calcified plaque components compared with reference standard reconstructions [53]. Adding spectral information could improve plaque quantification with VMI reconstructions. Nevertheless, standard protocols are needed to determine which VMIs are optimal for plaque volume assessment [54]. Through the integration of both high resolution and spectral information, PCD-CT facilitates the characterisation of plaque features, including lipid-rich necrotic cores, spotty calcifications and plaque ulceration. This detailed characterisation offers valuable insights into plaque vulnerability and the risk of rupture, crucial considerations in determining patient prognosis and enhancing treatment strategies, even in asymptomatic patients.

PCD-CT might come to play a role in epicardial adipose tissue (EAT) imaging. The density and extent of pericoronary EAT serve as a marker for vascular inflammation, which seems to have predictive value for adverse cardiac events [55]. Mergen et al. conducted a study assessing pericoronary EAT attenuation measurements with PCD-CT at different VMI energy levels in a phantom model [56]. VMI reconstructions at 70 keV offered the most precise estimates of fat attenuation when compared with conventional CT at 120 kV [56]. In a study by Risch et al., virtual non-contrast reconstructions derived from PCD-CT angiography datasets did provide consistent EAT volume measurements in comparison with true non-contrast series, allowing reduction in radiation dose and acquisition time [57]. These findings may aid in the standardisation of pericoronary EAT assessment.

Multiple studies have investigated the viability of myocardial perfusion CT, suggesting similar accuracy when compared with CMR perfusion imaging [58]. However, the assessment of iodine maps can be hindered due to beam hardening and other artefacts [58]. PCD-CT could prove advantageous by reducing these artefacts alongside its increased spatial and temporal resolution. Combining CT perfusion with CCTA and LE imaging in patients with intermediate stenoses, where haemodynamic significance is not certain, facilitates the evaluation of myocardial ischaemia, coronary anatomy and myocardial tissue viability in a single PCD-CT examination. This provides a one-stop-shop for making clinical decisions regarding revascularisation. In a case report, Polacin et al. detected hypodense myocardium with non-contrast PCD-CT and reduced iodine concentrations in the same region on dual-energy iodine maps from PCD-CT angiography images, corresponding with an ischaemic transmural scar visualised on LE_CMR_ [59].

In both pre-procedural and post-procedural imaging of valvular interventions, the use of PCD-CT could also become of interest. In pre-procedural planning, higher spatial resolutions and blooming artefact reduction allow reliable depiction of important anatomic structures. Specifically in the context of patients undergoing TAVR, integrating LE_PCD-CT_ into their pre-procedural scan protocol can even offer prognostic insights. Alongside their previously mentioned prognostic implications, pre-operative ECV measurements in TAVR patients can predict prognosis [60]. Furthermore, LE_PCD-CT_ can assist in the detection of concomitant cardiac amyloidosis in this population. Its identification carries prognostic implications and can impact treatment strategies [61]. On the other hand, in post-procedural imaging of valvular interventions, other advantages of PCD-CT are of value, such as metal artefact reduction and spectral reconstructions. These aspects can aid in the diagnostic dilemma regarding the increasing gradient in prosthetic valves by allowing more accurate assessment of the implanted valve and differentiation between post-procedural complications such as thrombus and pannus formation, bioprosthetic degenerative disease and endocarditis [62].

Another anticipated prospect arising from the application of PCD-CT technology is the potential for both contrast and radiation dose reduction. Research by Yu et al. revealed that the contrast-to-noise ratio of iodine contrast versus water increased by as much as 25% with PCD-CT when compared with conventional CT [63]. This implicates that reduced iodine concentrations in contrast media may suffice for adequate lumen visualisation. Furthermore, the enhanced contrast-to-noise ratio associated with PCD-CT can lead to a decrease in radiation dose. Several studies have examined this hypothesis and indicated reductions in radiation levels ranging from 30–60% [1]. The previously stated possible outcomes of PCD-CT do not solely improve image quality but could also aid in mitigating the risks associated with contrast and radiation exposure during clinical imaging procedures.

All the previously discussed possibilities and benefits for PCD-CT position it as a strong candidate for becoming the new standard; however, certain limitations associated with the use of PCD-CT in cardiac imaging are worth mentioning. Firstly, while registries can provide insights into the diagnostic performance of PCD-CT, large-scale clinical trials are necessary to establish its clinical utility and impact. These studies are crucial for investigating whether advanced technologies such as PCD-CT can reduce downstream testing, improve cost-effectiveness, and enhance patient outcomes through better risk stratification and personalised treatment approaches. Secondly, standardisation of imaging protocols and post-processing techniques among centres are essential to reproduce and compare findings. In addition, the availability of PCD-CT scanners is rather limited and costly as the production is still in the relatively early stages. When PCD technology becomes more established, production costs will likely decrease. Addressing cost-effectiveness is important for the implementation of PCD-CT in routine clinical practice.

Lastly, the expanding possibilities offered by PCD-CT highlight the need for increased collaboration between cardiovascular radiologists and imaging cardiologists. This collaboration is essential for identifying relevant indications, correct clinical interpretation of PCD-CT images and defining appropriate PCD-CT scan protocols.

## Conclusion

In conclusion, PCD-CT currently enhances diagnostic accuracy with its high spatial resolution, reduced calcium blooming, minimised artefacts, precise stenosis grading and stent imaging capabilities. These advancements lead to better risk stratification of patients. Looking ahead, tissue characterisation, especially through the integration of UHR imaging with spectral information, and both pre-procedural and post-procedural imaging, will become increasingly important. Furthermore, PCD-CT holds potential for reducing radiation doses and contrast media volume without compromising image quality, enhancing patient safety. This technology could reduce the necessity for invasive procedures and further testing, thereby improving patient outcomes and optimising healthcare resource utilisation.

## References

[1] Willemink MJ, Persson M, Pourmorteza A, Pelc NJ, Fleischmann D. Photon-counting CT: technical principles and clinical prospects. Radiology. 2018;289:293–312. 30179101 10.1148/radiol.2018172656

[2] van der Bie J, van Straten M, Booij R, et al. Photon-counting CT: review of initial clinical results. Eur J Radiol. 2023;163:110829. 37080060 10.1016/j.ejrad.2023.110829

[3] Douek PC, Boccalini S, Oei EHG, et al. Clinical applications of photon-counting CT: a review of pioneer studies and a glimpse into the future. Radiology. 2023;309:e222432. 37787672 10.1148/radiol.222432 PMC10623209

[4] Flohr T, Schmidt B. Technical basics and clinical benefits of photon-counting CT. Invest Radiol. 2023;58:441–50. 37185302 10.1097/RLI.0000000000000980 PMC10259209

[5] Nakamura Y, Higaki T, Kondo S, Kawashita I, Takahashi I, Awai K. An introduction to photon-counting detector CT (PCD CT) for radiologists. Jpn J Radiol. 2023;41:266–82. 36255601 10.1007/s11604-022-01350-6 PMC9974724

[6] Danad I, Fayad ZA, Willemink MJ, Min JK. New applications of cardiac computed tomography: dual-energy, spectral, and molecular CT imaging. JACC Cardiovasc Imaging. 2015;8:710–23. 26068288 10.1016/j.jcmg.2015.03.005 PMC4467470

[7] Si-Mohamed SA, Boccalini S, Lacombe H, et al. Coronary CT angiography with photon-counting CT: first-in-human results. Radiology. 2022;303:303–13. 35166583 10.1148/radiol.211780

[8] Holmes TW, Yin Z, Stevens GM, et al. Ultra-high-resolution spectral silicon-based photon-counting detector CT for coronary CT angiography: Initial results in a dynamic phantom. J Cardiovasc Comput Tomogr. 2023;17:341–4. 37567802 10.1016/j.jcct.2023.08.003

[9] Zhan X, Zhang R, Niu X, et al. Comprehensive evaluations of a prototype full field-of-view photon counting CT system through phantom studies. Phys Med Biol. 2023; 10.1088/1361-6560/acebb3. 37506710 10.1088/1361-6560/acebb3

[10] Greenland P, Blaha MJ, Budoff MJ, Erbel R, Watson KE. Coronary calcium score and cardiovascular risk. J Am Coll Cardiol. 2018;72:434–47. 30025580 10.1016/j.jacc.2018.05.027 PMC6056023

[11] Knuuti J, Wijns W, Saraste A, et al. 2019 ESC guidelines for the diagnosis and management of chronic coronary syndromes. Eur Heart J. 2020;41:407–77. 31504439 10.1093/eurheartj/ehz425

[12] van der Werf NR, Si-Mohamed S, Rodesch PA, et al. Coronary calcium scoring potential of large field-of-view spectral photon-counting CT: a phantom study. Eur Radiol. 2022;32:152–62. 34255159 10.1007/s00330-021-08152-w PMC8660747

[13] Gassert FG, Schacky CE, Müller-Leisse C, et al. Calcium scoring using virtual non-contrast images from a dual-layer spectral detector CT: comparison to true non-contrast data and evaluation of proportionality factor in a large patient collective. Eur Radiol. 2021;31:6193–9. 33474570 10.1007/s00330-020-07677-w PMC8270810

[14] Song I, Yi JG, Park JH, Kim SM, Lee KS, Chung MJ. Virtual non-contrast CT using dual-energy spectral CT: feasibility of coronary artery calcium scoring. Korean J Radiol. 2016;17:321–9. 27134521 10.3348/kjr.2016.17.3.321 PMC4842852

[15] Schwarz F, Nance JW Jr., Ruzsics B, Bastarrika G, Sterzik A, Schoepf UJ. Quantification of coronary artery calcium on the basis of dual-energy coronary CT angiography. Radiology. 2012;264:700–7. 22829684 10.1148/radiol.12112455

[16] Emrich T, Aquino G, Schoepf UJ, et al. Coronary computed tomography angiography-based calcium scoring: in vitro and in vivo validation of a novel virtual noniodine reconstruction algorithm on a clinical, first-generation dual-source photon counting-detector system. Invest Radiol. 2022;57:536–43. 35318969 10.1097/RLI.0000000000000868

[17] Sharma SP, van der Bie J, van Straten M, et al. Coronary calcium scoring on virtual non-contrast and virtual non-iodine reconstructions compared to true non-contrast images using photon-counting computed tomography. Eur Radiol. 2023;34:3699–707. 37940711 10.1007/s00330-023-10402-y PMC11166815

[18] Danad I, Szymonifka J, Twisk JWR, et al. Diagnostic performance of cardiac imaging methods to diagnose ischaemia-causing coronary artery disease when directly compared with fractional flow reserve as a reference standard: a meta-analysis. Eur Heart J. 2017;38:991–8. 27141095 10.1093/eurheartj/ehw095 PMC5381594

[19] Mowatt G, Cook JA, Hillis GS, et al. 64-Slice computed tomography angiography in the diagnosis and assessment of coronary artery disease: systematic review and meta-analysis. Heart. 2008;94:1386–93. 18669550 10.1136/hrt.2008.145292

[20] van der Bie J, Sharma SP, van Straten M, et al. Image quality assessment of coronary artery segments using ultra-high resolution dual source photon-counting detector computed tomography. Eur J Radiol. 2024;171:111282. 38190778 10.1016/j.ejrad.2023.111282

[21] Mergen V, Sartoretti T, Baer-Beck M, et al. Ultra-high-resolution coronary CT angiography with photon-counting detector CT: feasibility and image characterization. Invest Radiol. 2022;57:780–8. 35640019 10.1097/RLI.0000000000000897 PMC10184822

[22] Zsarnoczay E, Fink N, Schoepf UJ, et al. Ultra-high resolution photon-counting coronary CT angiography improves coronary stenosis quantification over a wide range of heart rates—a dynamic phantom study. Eur J Radiol. 2023;161:110746. 36821957 10.1016/j.ejrad.2023.110746

[23] Koons EK, Rajiah PS, Thorne JE, et al. Coronary artery stenosis quantification in patients with dense calcifications using ultra-high-resolution photon-counting-detector computed tomography. J Cardiovasc Comput Tomogr. 2023;18:56–61. 37945454 10.1016/j.jcct.2023.10.009 PMC10922101

[24] McCollough CH, Rajiah P, Bois JP, et al. Comparison of photon-counting detector and energy-integrating detector CT for visual estimation of coronary percent luminal stenosis. Radiology. 2023;309:e230853. 38051190 10.1148/radiol.230853 PMC10741385

[25] Nishihara T, Miyoshi T, Nakashima M, et al. Diagnostic improvements of calcium-removal image reconstruction algorithm using photon-counting detector CT for calcified coronary lesions. Eur J Radiol. 2024;172:111354. 38309215 10.1016/j.ejrad.2024.111354

[26] Hagar MT, Soschynski M, Saffar R, et al. Accuracy of ultrahigh-resolution photon-counting CT for detecting coronary artery disease in a high-risk population. Radiology. 2023;307:e223305. 37338354 10.1148/radiol.223305

[27] Brendel JM, Walterspiel J, Hagen F, et al. Coronary artery disease evaluation during transcatheter aortic valve replacement work-up using photon-counting CT and artificial intelligence. Diagn Interv Imaging. 2024;105:273–80. 38368176 10.1016/j.diii.2024.01.010

[28] Zsarnoczay E, Pinos D, Schoepf UJ, et al. Intra-individual comparison of coronary CT angiography-based FFR between energy-integrating and photon-counting detector CT systems. Int J Cardiol. 2024;399:131684. 38151162 10.1016/j.ijcard.2023.131684

[29] Sun Z, Almutairi AM. Diagnostic accuracy of 64 multislice CT angiography in the assessment of coronary in-stent restenosis: a meta-analysis. Eur J Radiol. 2010;73:266–73. 19056191 10.1016/j.ejrad.2008.10.025

[30] Boccalini S, Si-Mohamed SA, Lacombe H, et al. First in-human results of computed tomography angiography for coronary stent assessment with a spectral photon counting computed tomography. Invest Radiol. 2022;57:212–21. 34711766 10.1097/RLI.0000000000000835 PMC8903215

[31] Decker JA, O’Doherty J, Schoepf UJ, et al. Stent imaging on a clinical dual-source photon-counting detector CT system-impact of luminal attenuation and sharp kernels on lumen visibility. Eur Radiol. 2023;33:2469–77. 36462045 10.1007/s00330-022-09283-4

[32] Hagar MT, Soschynski M, Saffar R, et al. Ultra-high-resolution photon-counting detector CT in evaluating coronary stent patency: a comparison to invasive coronary angiography. Eur Radiol. 2024;34:4273–83. 38177617 10.1007/s00330-023-10516-3 PMC11213791

[33] Kim RJ, Wu E, Rafael A, et al. The use of contrast-enhanced magnetic resonance imaging to identify reversible myocardial dysfunction. N Engl J Med. 2000;343:1445–53. 11078769 10.1056/NEJM200011163432003

[34] Mahrholdt H, Wagner A, Judd RM, Sechtem U, Kim RJ. Delayed enhancement cardiovascular magnetic resonance assessment of non-ischaemic cardiomyopathies. Eur Heart J. 2005;26:1461–74. 15831557 10.1093/eurheartj/ehi258

[35] Mewton N, Liu CY, Croisille P, Bluemke D, Lima JA. Assessment of myocardial fibrosis with cardiovascular magnetic resonance. J Am Coll Cardiol. 2011;57:891–903. 21329834 10.1016/j.jacc.2010.11.013 PMC3081658

[36] Parsai C, O’Hanlon R, Prasad SK, Mohiaddin RH. Diagnostic and prognostic value of cardiovascular magnetic resonance in non-ischaemic cardiomyopathies. J Cardiovasc Magn Reson. 2012;14:54. 22857649 10.1186/1532-429X-14-54 PMC3436728

[37] Messroghli DR, Moon JC, Ferreira VM, et al. Clinical recommendations for cardiovascular magnetic resonance mapping of T1, T2, T2* and extracellular volume: a consensus statement by the society for cardiovascular magnetic resonance (SCMR) endorsed by the European association for cardiovascular imaging (EACVI). J Cardiovasc Magn Reson. 2017;19:75. 28992817 10.1186/s12968-017-0389-8 PMC5633041

[38] Arbelo E, Protonotarios A, Gimeno JR, et al. ESC guidelines for the management of cardiomyopathies. Eur Heart J. 2023;44:3503–626. 37622657 10.1093/eurheartj/ehad194

[39] Andreini D, Conte E, Mushtaq S, et al. Comprehensive evaluation of left ventricle dysfunction by a new computed tomography scanner: the E‑PLURIBUS study. JACC Cardiovasc Imaging. 2023;16:175–88. 36444769 10.1016/j.jcmg.2022.08.005

[40] Oyama-Manabe N, Oda S, Ohta Y, Takagi H, Kitagawa K, Jinzaki M. Myocardial late enhancement and extracellular volume with single-energy, dual-energy, and photon-counting computed tomography. J Cardiovasc Comput Tomogr. 2024;18:3–10. 38218665 10.1016/j.jcct.2023.12.006

[41] Scully PR, Bastarrika G, Moon JC, Treibel TA. Myocardial extracellular volume quantification by cardiovascular magnetic resonance and computed tomography. Curr Cardiol Rep. 2018;20:15. 29511861 10.1007/s11886-018-0961-3 PMC5840231

[42] Sartoretti T, Wildberger JE, Flohr T, Alkadhi H. Photon-counting detector CT: early clinical experience review. Br J Radiol. 2023;96:20220544. 36744809 10.1259/bjr.20220544 PMC10321251

[43] Nishihara T, Miyoshi T, Akagi N, Nakashima M, Nakamura K, Ito H. Myocardial late iodine enhancement using photon-counting computed tomography in patients with hypertrophic cardiomyopathy. Circ Rep. 2023;5:269–70. 37305798 10.1253/circrep.CR-23-0034 PMC10247347

[44] Symons R, Krauss B, Sahbaee P, et al. Photon-counting CT for simultaneous imaging of multiple contrast agents in the abdomen: an in vivo study. Med Phys. 2017;44:5120–7. 28444761 10.1002/mp.12301 PMC5699215

[45] Symons R, Cork TE, Lakshmanan MN, et al. Dual-contrast agent photon-counting computed tomography of the heart: initial experience. Int J Cardiovasc Imaging. 2017;33:1253–61. 28289990 10.1007/s10554-017-1104-4

[46] Zhang H, Guo H, Liu G, et al. CT for the evaluation of myocardial extracellular volume with MRI as reference: a systematic review and meta-analysis. Eur Radiol. 2023;33:8464–76. 37378712 10.1007/s00330-023-09872-x

[47] Yang EY, Ghosn MG, Khan MA, et al. Myocardial extracellular volume fraction adds prognostic information beyond myocardial replacement fibrosis. Circ Cardiovasc Imaging. 2019;12:e9535. 31838882 10.1161/CIRCIMAGING.119.009535 PMC7529265

[48] Koike H, Fukui M, Treibel T, et al. Comprehensive myocardial assessment by computed tomography: impact on short-term outcomes after transcatheter aortic valve replacement. JACC Cardiovasc Imaging. 2023;17:396–407. 37921717 10.1016/j.jcmg.2023.08.008

[49] Deux JF, Nouri R, Tacher V, et al. Diagnostic value of extracellular volume quantification and myocardial perfusion analysis at CT in cardiac amyloidosis. Radiology. 2021;300:326–35. 34100681 10.1148/radiol.2021204192

[50] Yashima S, Takaoka H, Iwahana T, et al. Evaluation of extracellular volume by computed tomography is useful for prediction of prognosis in dilated cardiomyopathy. Heart Vessels. 2023;38:185–94. 35963911 10.1007/s00380-022-02154-4

[51] Mergen V, Sartoretti T, Klotz E, et al. Extracellular volume quantification with cardiac late enhancement scanning using dual-source photon-counting detector CT. Invest Radiol. 2022;57:406–11. 35066531 10.1097/RLI.0000000000000851 PMC9390230

[52] Aquino GJ, O’Doherty J, Schoepf UJ, et al. Myocardial characterization with extracellular volume mapping with a first-generation photon-counting detector CT with MRI reference. Radiology. 2023;307:e222030. 36719292 10.1148/radiol.222030

[53] Mergen V, Eberhard M, Manka R, Euler A, Alkadhi H. First in-human quantitative plaque characterization with ultra-high resolution coronary photon-counting CT angiography. Front Cardiovasc Med. 2022;9:981012. 36148053 10.3389/fcvm.2022.981012 PMC9485480

[54] Vattay B, Szilveszter B, Boussoussou M, et al. Impact of virtual monoenergetic levels on coronary plaque volume components using photon-counting computed tomography. Eur Radiol. 2023;33:8528–39. 37488295 10.1007/s00330-023-09876-7 PMC10667372

[55] Chong B, Jayabaskaran J, Ruban J, et al. Epicardial adipose tissue assessed by computed tomography and echocardiography are associated with adverse cardiovascular outcomes: a systematic review and meta-analysis. Circ Cardiovasc Imaging. 2023;16:e15159. 37192298 10.1161/CIRCIMAGING.122.015159

[56] Mergen V, Ried E, Allmendinger T, et al. Epicardial adipose tissue attenuation and fat attenuation index: phantom study and in vivo measurements with photon-counting detector CT. AJR Am J Roentgenol. 2022;218:822–9. 34877869 10.2214/AJR.21.26930

[57] Risch F, Schwarz F, Braun F, et al. Assessment of epicardial adipose tissue on virtual non-contrast images derived from photon-counting detector coronary CTA datasets. Eur Radiol. 2023;33:2450–60. 36462042 10.1007/s00330-022-09257-6 PMC10017616

[58] Varga-Szemes A, Meinel FG, De Cecco CN, Fuller SR, Bayer RR 2nd, Schoepf UJ. CT myocardial perfusion imaging. AJR Am J Roentgenol. 2015;204:487–97. 25714277 10.2214/AJR.14.13546

[59] Polacin M, Templin C, Manka R, Alkadhi H. Photon-counting computed tomography for the diagnosis of myocardial infarction with non-obstructive coronary artery disease. Eur Heart J Case Rep. 2022;6:ytac28. 35233486 10.1093/ehjcr/ytac028 PMC8874819

[60] Vignale D, Palmisano A, Gnasso C, et al. Extracellular volume fraction (ECV) derived from pre-operative computed tomography predicts prognosis in patients undergoing transcatheter aortic valve implantation (TAVI). Eur Heart J Cardiovasc Imaging. 2023;24:887–96. 36916015 10.1093/ehjci/jead040 PMC10284054

[61] Jaiswal V, Agrawal V, Khulbe Y, et al. Cardiac amyloidosis and aortic stenosis: a state-of-the-art review. Eur Heart J Open. 2023;3:oead106. 37941729 10.1093/ehjopen/oead106 PMC10630099

[62] van der Bie J, Sharma SP, van Straten M, et al. Photon-counting detector CT in patients pre- and post-transcatheter aortic valve replacement. Radiol Cardiothorac Imaging. 2023;5:e220318. 37124634 10.1148/ryct.220318 PMC10141309

[63] Yu Z, Leng S, Jorgensen SM, et al. Evaluation of conventional imaging performance in a research whole-body CT system with a photon-counting detector array. Phys Med Biol. 2016;61:1572–95. 26835839 10.1088/0031-9155/61/4/1572 PMC4782185

